# MECHANISMS OF LONGEVITY: LESSONS FROM LONG-LIVED MAMMALS

**DOI:** 10.1093/geroni/igz038.026

**Published:** 2019-11-08

**Authors:** Vera Gorbunova

**Affiliations:** University of Rochester, Rochester, New York, United States

## Abstract

Animals have evolved a dramatic diversity of aging rates with lifespans ranging from 2 years to 200 years. This natural diversity of lifespan can be exploited to understand the mechanisms of longevity and develop anti-aging interventions. Our goal is to identify mechanisms that allow such exceptionally long-lived animals to live long and healthy lives and then use these mechanisms to benefit human health. Naked mole rat is the longest-lived rodent with the maximum lifespan of 32 years. We discovered that the mechanism of longevity and cancer resistance in the naked mole rat mediated by high molecular weight hyaluronan. I will discuss the mouse model we generated that mimics the naked mole rat and shows increased healthspan and lifespan. I will also the role of SIRT6 in mediating longevity across mammals and in human centenarians by improving DNA repair and silencing transposable elements.

